# Response to comment on 'Palovarotene reduces heterotopic ossification in juvenile FOP mice but exhibits pronounced skeletal toxicity'

**DOI:** 10.7554/eLife.43928

**Published:** 2019-01-30

**Authors:** David J Goldhamer, John B Lees-Shepard

**Affiliations:** Department of Molecular and Cell BiologyUniversity of ConnecticutStorrsUnited States; Maine Medical Center Research InstituteUnited States; University of OxfordUnited Kingdom

**Keywords:** Fibrodysplasia ossificans progressiva, Palovarotene, RARγ agonist, heterotopic ossification, skeletal toxicity, growth plate, Mouse

## Abstract

We respond to concerns expressed by Pacifici and Shore (2019) about a recent paper (Lees-Shepard and Goldhamer, 2018a) in which we reported that the drug palovarotene can have severe side effects in a mouse model of fibrodysplasia ossificans progressiva.

## Introduction

We recently published a study that demonstrated the toxic effects of palovarotene on the skeletons of juvenile mice (Lees-Shepard et al., 2018a). Pacifici and Shore have raised concerns about this study (Pacifici and Shore, 2019): while they do not challenge the veracity of the results or the rigor with which the experiments were performed, they question the relevance of our study to the ongoing efficacy and safety study of palovarotene for the treatment of fibrodysplasia ossificans progressiva (FOP) being conducted by Clementia Pharmaceuticals (https://clinicaltrials.gov/ct2/show/NCT03312634). Here we respond to their concerns and provide context for the experimental design of our study (which was supported by a sponsored research agreement with Clementia Pharmaceuticals).

## Discussion

### Route of palovarotene administration

Pacifici and Shore strongly imply that the goal of our study was to inform the development of acceptable safety parameters for the use of palovarotene in juvenile FOP patients, and commented that the method we used for drug delivery (intraperitoneal injection: IP) is never used in patients. It will be clear to readers, however, that the comment in the introduction of our paper concerning an "acceptable safety profile" was a general comment relevant to all potential therapies for FOP because of the prevalence of heterotopic ossification (HO) in juvenile patients. A comprehensive analysis of palovarotene toxicity was neither the stated goal nor the intent of our publication.

IP administration of palovarotene was considered necessary for our study to avoid the complication of oral dosing of animals with jaw HO, which is highly penetrant in our model, and to eliminate the real risk of inducing HO in throat and jaw muscles by daily manipulation and probable irritation. No published studies have directly compared palovarotene C_max_ values for IP and oral dosing, and Pacifici and Shore provide no evidence that cell and tissue levels of palovarotene are affected by route of administration. Further, regardless of possible differences in C_max_, it is not at all clear that C_max_ is the most relevant pharmacokinetic parameter.

### FOP mouse model

Pacifici and Shore also criticize our use of a Pdgfrα-Cre driver to conditionally recombine the FOP allele, *Acvr1^tnR206H^*, stating that our mouse model "does not mimic FOP patients". While it is true that Pdgfrα-Cre;*Acvr1^tnR206H/+^* mice differ from FOP in humans in that the mutation in this mouse model is restricted to cells that express, or have expressed, Pdgfrα, this restriction was an essential feature of our study. The study had two primary objectives: i) to examine the effect of palovarotene on HO driven by fibro/adipogenic progenitors; ii) to assess the efficacy of palovarotene in inhibiting body-wide spontaneous HO. As Pdgfrα is the best known single marker for fibro/adipogenic progenitors (reviewed in Lees-Shepard and Goldhamer, 2018), and because Pdgfrα-Cre;*Acvr1^tnR206H/+^* mice recapitulate the major pathogenic manifestations of FOP, including progressive HO at all major anatomical sites affected in FOP patients (Lees-Shepard et al., 2018a; Lees-Shepard et al., 2018b), the Pdgfrα-Cre driver was ideally suited for our studies. We also note that no additional cell types need to be invoked to explain the full repertoire of HO in FOP (Lees-Shepard et al., 2018b).

In 2016, Pacifici, Shore and colleagues used juvenile Prrx-Cre;*Acvr1^R206H^* mice to test the safety and efficacy of palovarotene (Chakkalakal et al., 2016). Prrx-Cre is broadly expressed in limb mesenchyme during development, and it is reasonable to assume that the large majority of cells in the limbs of these mice had undergone Cre-recombination at the *Acvr1^R206H^* locus. However, this similarity in genetic make-up does not necessarily mean that Prrx-Cre;*Acvr1^R206H^* mice are a better model of FOP in humans, as Pacifici and Shore contend. Indeed, while FOP patients exhibit only minor developmental abnormalities (most notably, great toe malformations), mice carrying the *Acvr1^R206H^* mutation in all cells exhibit severe skeletal developmental defects and die perinatally (Chakkalakal et al., 2012; Kaplan et al., 2012). Although the reasons for this dramatic phenotypic difference between mice and humans are unknown, it is clear that genetic status of the *Acvr1* gene is only one of potentially many model parameters to consider, depending on study goals.

### Effects on growth plates and joints

Our publication documented severe skeletotoxic effects of palovarotene on both wild-type and Pdgfrα-Cre;*Acvr1^tnR206H/+^* mice, including loss of tibial growth plates (Lees-Shepard et al., 2018a). In contrast, Chakkalakal et al. (2016) showed that palovarotene treatment actually improved growth plate morphology in Prrx-Cre;*Acvr1^R206H^* mice and had only slight effects on skeletal tissues of wild-type controls. They attributed the improvement in Prrx-Cre;*Acvr1^R206H^* mice to the offsetting actions of increased BMP signaling resulting from *Acvr1^R206H^* expression in growth plate cartilage and the inhibitory effects of palovarotene on BMP signaling. Pacifici and Shore argue that the deleterious effects of palovarotene on growth plates of Pdgfrα-Cre;*Acvr1^tnR206H/+^* mice resulted from the lack of *Acvr1^R206H^* expression. At present, this hypothesis remains speculative. Pacifici and Shore assume that the growth plates in our model do not express *Acvr1^R206H^*, stating: "Given that the Pdgfrα gene is not known to be expressed in chondrocytes (Hamilton et al., 2003), it is nearly certain that growth plate and articular chondrocytes were not targeted by Pdgfrα-Cre and remained largely, if not totally, wild-type.’" However, Pdgfrα expression has been well-documented in limb mesenchyme, including pre-cartilage mesenchyme (Ataliotis, 2000; Orr-Urtreger and Lonai, 1992; Schatteman et al., 1992), so it would not be surprising if the *Acvr1^tnR206H^* allele was recombined in the growth plates of Pdgfrα-Cre;*Acvr1^tnR206H/+^* mice. In fact, the entire premise of using Prrx-Cre mice in their study is that broad, antecedent expression of Cre in embryonic limb mesenchyme should result in juvenile limbs in which most or all cells carry the *Acvr1^R206H^* mutation. Neither we nor Chakkalakal et al. (2016) assessed *Acvr1^R206H^* expression in growth plate cartilage, and this would certainly be a worthwhile area of investigation.

Another issue, not discussed by Pacifici and Shore, is that Chakkalakal et al. (2016) used a distinct dosing regimen (which does not resemble that used in the FOP clinical trial) and earlier study endpoint. Specifically, Prrx-Cre;*Acvr1^R206H^* mice were dosed for the first 15 days of life by daily administration of palovarotene to lactating mothers, followed by direct administration by oral gavage from P16 to P30, using an every-other-day dosing schedule and without adjusting for body weight. To begin to address the reasons for the markedly different effects of palovarotene reported by Chakkalakal et al. (2016) and Lees-Shepard et al. (2018a), we conducted an additional experiment in which juvenile mice were treated with palovarotene by IP administration (as in Lees-Shepard et al., 2018a), but with the dosing regimen and 30 day endpoint used by Chakkalakal et al. (2016). Notably, we did not observe loss of growth plates in either wild-type (n = 4) or Pdgfrα-Cre;*Acvr1^tnR206H/+^* mice (n = 4) (unpublished observations), consistent with the results of Chakkalakal et al. (2016). These results suggest that dosing regimen and/or study endpoint may explain the disparate study outcomes. As such, attributing the differences to the route of administration or the FOP mouse model used is, at best, premature.

### Conclusion

The criticisms of our *eLife* publication by Pacifici and Shore are related to its relevance to ongoing clinical programs. However, these criticisms are based on a series of assumptions that have not been experimentally demonstrated. All mouse studies should be interpreted cautiously, and translation of individual studies to humans should not be assumed or implied. Our work should not be interpreted to mean that palovarotene will necessarily result in skeletal toxicity in children with FOP. Similarly, we urge caution when extrapolating the safety and efficacy data of Chakkalakal et al. (2016) to the ongoing clinical study of palovarotene for the treatment of FOP in children.

## References

[bib1] Ataliotis P (2000). Platelet-derived growth factor A modulates limb chondrogenesis both in vivo and in vitro. Mechanisms of Development.

[bib2] Chakkalakal SA, Zhang D, Culbert AL, Convente MR, Caron RJ, Wright AC, Maidment AD, Kaplan FS, Shore EM (2012). An Acvr1 R206H knock-in mouse has fibrodysplasia ossificans progressiva. Journal of Bone and Mineral Research.

[bib3] Chakkalakal SA, Uchibe K, Convente MR, Zhang D, Economides AN, Kaplan FS, Pacifici M, Iwamoto M, Shore EM (2016). Palovarotene inhibits heterotopic ossification and maintains limb mobility and growth in mice with the human ACVR1(R206H) Fibrodysplasia ossificans progressiva (FOP) Mutation. Journal of Bone and Mineral Research.

[bib4] Hamilton TG, Klinghoffer RA, Corrin PD, Soriano P (2003). Evolutionary divergence of platelet-derived growth factor alpha receptor signaling mechanisms. Molecular and Cellular Biology.

[bib5] Kaplan FS, Chakkalakal SA, Shore EM (2012). Fibrodysplasia ossificans progressiva: mechanisms and models of skeletal metamorphosis. Disease Models & Mechanisms.

[bib6] Lees-Shepard JB, Nicholas SE, Stoessel SJ, Devarakonda PM, Schneider MJ, Yamamoto M, Goldhamer DJ (2018a). Palovarotene reduces heterotopic ossification in juvenile FOP mice but exhibits pronounced skeletal toxicity. eLife.

[bib7] Lees-Shepard JB, Yamamoto M, Biswas AA, Stoessel SJ, Nicholas SE, Cogswell CA, Devarakonda PM, Schneider MJ, Cummins SM, Legendre NP, Yamamoto S, Kaartinen V, Hunter JW, Goldhamer DJ (2018b). Activin-dependent signaling in fibro/adipogenic progenitors causes fibrodysplasia ossificans progressiva. Nature Communications.

[bib8] Lees-Shepard JB, Goldhamer DJ (2018). Stem cells and heterotopic ossification: Lessons from animal models. Bone.

[bib9] Orr-Urtreger A, Lonai P (1992). Platelet-derived growth factor-A and its receptor are expressed in separate, but adjacent cell layers of the mouse embryo. Development.

[bib10] Pacifici M, Shore EM (2019). Comment on 'Palovarotene reducesheterotopic ossification in juvenile FOP mice but exhibits pronounced skeletaltoxicity'. eLife.

[bib11] Schatteman GC, Morrison-Graham K, van Koppen A, Weston JA, Bowen-Pope DF (1992). Regulation and role of PDGF receptor alpha-subunit expression during embryogenesis. Development.

